# A case of pregnant patient with stenotic bicuspid aortic valve

**DOI:** 10.1002/ccr3.8104

**Published:** 2023-12-19

**Authors:** Joanna Lewek, Marek Maciejewski, Maciej Banach, Agata Magdalena Bielecka‐Dąbrowa

**Affiliations:** ^1^ Department of Preventive Cardiology and Lipidology, Chair of Nephrology and Hypertension Medical University of Lodz Lodz Poland; ^2^ Department of Cardiology and Congenital Diseases of Adults Polish Mother's Memorial Hospital Research Institute (PMMHRI) Lodz Poland; ^3^ Cardiovascular Research Centre Zielona Góra Poland

**Keywords:** aortic stenosis, bicuspid aortic valve, interventional cardiology, pregnancy

## Abstract

**Key Clinical Message:**

Asymptomatic severe aortic stenosis (AS) during pregnancy remains challenging; however, the postponement of surgery with the possibility of valvuloplasty as a bridge therapy seems reasonable. Our case showed that despite physiological changes during pregnancy, the aortic valve defect did not worsen, which allowed us to avoid dilemmas related to anticoagulation on artificial valve.

**Abstract:**

A 31‐year‐old woman, with a bicuspid aortic aorta, post‐aortic valvulotomy, was listed for cardiac surgery because of severe aortic stenosis. However, the operation was postponed due to procreation plans. During the pregnancy and delivery, we did not observe neither deterioration of symptoms nor changes of echocardiographic parameters. Subsequent monthly echocardiographic studies did not reveal a significant increase of peak and mean aortic gradient.

Presented case reports showed that despite physiological changes associated with pregnancy, the aortic valve defect did not worsen, which allowed to avoid dilemmas related to anticoagulation on artificial valves.

## Graphical abstract

Peak aortic velocity 5 m/s with peak gradient 102 mmHg in pregnant patient with a bicuspid aorta post valvulotomy.

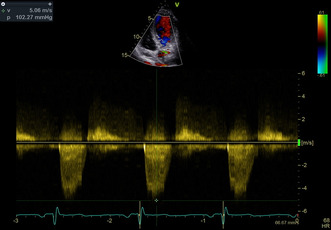

## 1 INTRODUCTION

Bicuspid aortic valve (BAV) remains the most common congenital heart disease considered to affect approximately 1% of the population with male‐to‐female ratio of two to one (0.5%–1.4% based on small echocardiography and autopsy studies) frequently leading to stenosis or incompetence of the valve in a relatively young age.^1^ Natural history of BAV degeneration and calcification is not established and differs among patients, ranging from severe stenosis in infancy to asymptomatic course in old age. Another issue are the physiological changes in cardiac hemodynamics during pregnancy including the increase of the plasma volume, elevation of heart rate as well as concomitant decrease of the systemic vascular resistance. Therefore, in the setting of a severely stenotic valve all of the mentioned above can cause decompensation. All symptomatic patients presenting with severe aortic stenosis (AS) or asymptomatic patients with echocardiographic images of decreased left ventricular ejection fraction (LVEF < 50%) or with an abnormal result of an exercise test should be advised against pregnancy. In such patients, surgery should be counseled pre‐pregnancy.^2^ We are lacking data about the treatment of asymptomatic severe AS or patients with no high‐risk features during stress testing before planned pregnancy, which constitutes a small realm of patients.

## 2 CASE REPORT

A 31‐year‐old woman, with a bicuspid aortic valve, post‐aortic valvulotomy (at the age of one year due to severe aortic stenosis), and hypothyroidism was admitted to the Department of Cardiology and Adult Congenital Diseases for diagnostics and determination of further procedures. There was no family history of cardiovascular diseases particularly congenital heart disturbances. The patient did not report any symptoms and therefore was not taking any medications besides levothyroxine, a synthetic form of the human hormone thyroxine due to hypothyroidism. She presented good exercise tolerance. Physical examination revealed a high‐pitched, crescendo‐decrescendo midsystolic ejection murmur that was heard best at the right upper sternal border and was radiating to the neck and carotid arteries. Heart rate was 70 beats per minute, and blood pressure was 110/64 mmHg on the left arm and 130/71 mmHg on the right arm. Besides abnormal values of thyroid hormones, she presented no other abnormalities in laboratory tests results. Body Mass Index (BMI) measured before pregnancy was at the lower limit of normal (BMI 20.20 kg/m^2^).

**FIGURE 1:**
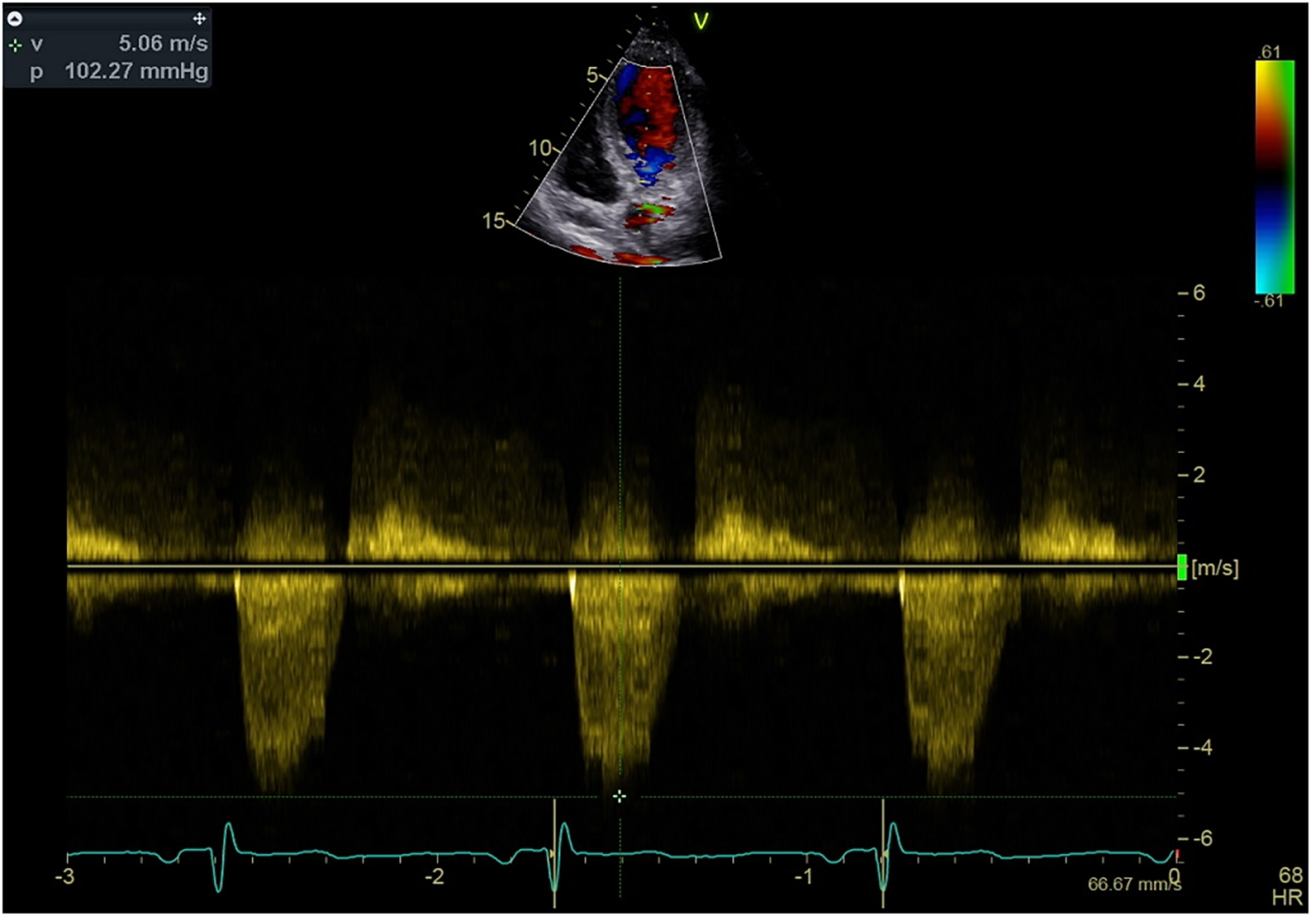
Peak aortic velocity 5 m/s with peak gradient 102 mmHg and mean gradient 60 mmHg.

**FIGURE 2:**
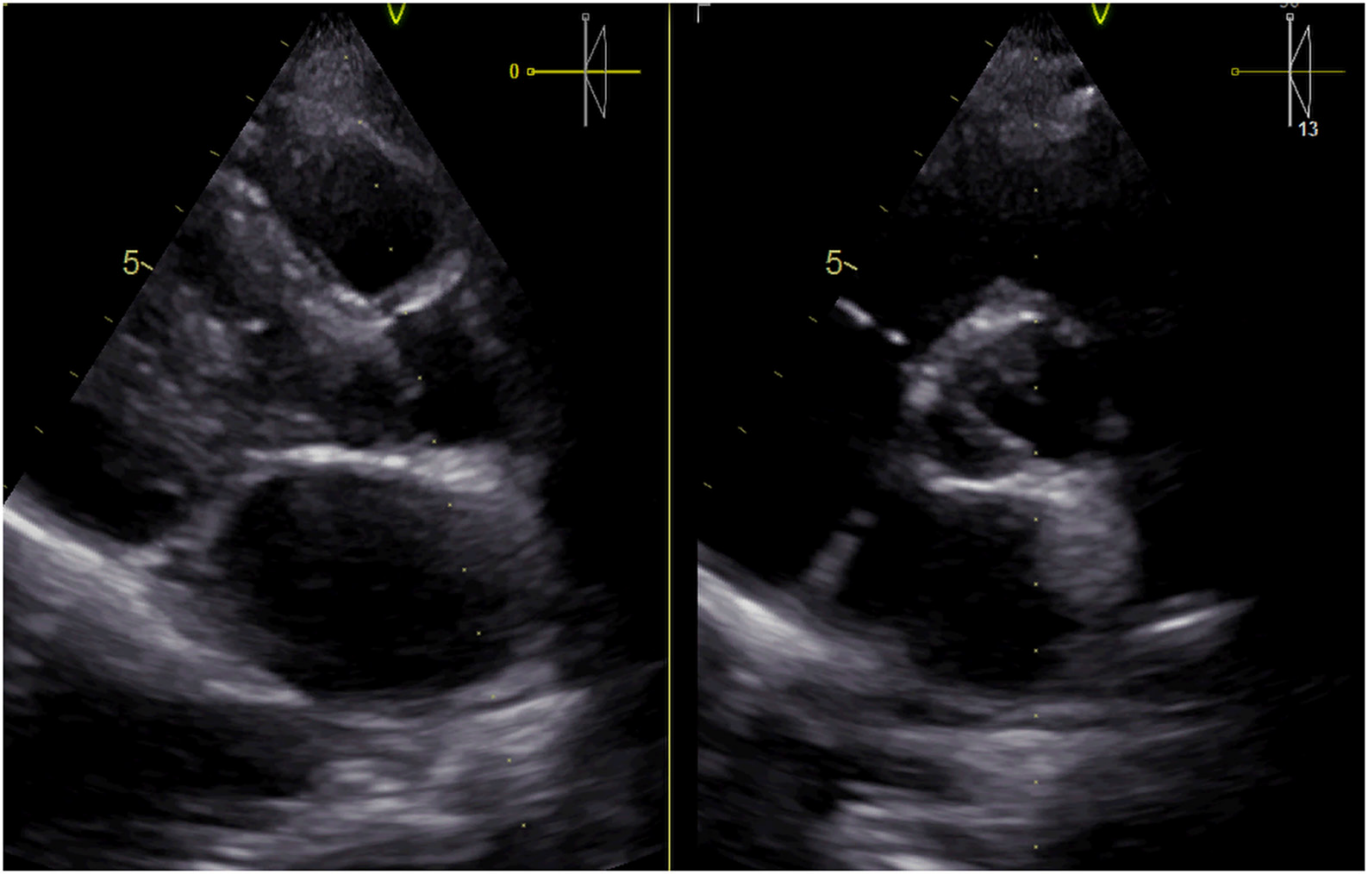
Aortic valve area planimetry from transesophageal echocardiography.

Transthoracic echocardiography showed the preserved systolic function of a mildly hypertrophic left ventricle with ejection fraction 65%, with no segmental abnormalities in wall contractility. In addition, a complex defect of a bicuspid aortic valve was visualized with severe stenosis (peak aortic gradient 102 mmHg, mean gradient 60 mmHg, valve area calculated with velocity time integral – indexed Aortic valve area – AVAi 0.4 cm^2^/m^2^) and moderate regurgitation (pressure half‐time – PHT was 381 ms) (Figures 1 and 2). Aortic dimensions were within normal range. Transesophageal echocardiography confirmed severe stenosis of aortic valve with AVAi 0.4 cm^2^/m^2^. The 24‐hour electrocardiography monitoring recorded a sinus rhythm with a tendency to sinus bradycardia (with average heart rate of 59/min) and single premature supraventricular beats. Mean blood pressure was 117/60 mmHg with a nighttime dipper profile in the 24‐hour ambulatory blood pressure monitoring. Chest angio Computer tomography, which was performed to check whether there is no aortopathy, showed thoracic aorta dimensions within normal range: ascending aorta, transverse dimensions of 19 mm, aortic arch 21 mm, descending aorta of 20 mm, and aorta in the supra‐diaphragmatic segment of 16 mm. Mild stenosis of the left subclavian artery was visualized. Exercise stress test was performed to check whether the patient is really asymptomatic, showing very good tolerance of exercise with 13.5 METs, clinically and electrocardiographically negative. No rhythm disturbances were observed. The blood pressure response was normal.

Based on the overall clinical picture, due to the procreative age of the patient, she was consulted with the Heart Team; further conservative treatment was recommended, with the possibility of becoming pregnant in the coming months, with strict cardiological, gynecological, and obstetric control. In cases of deterioration of the patient's condition, balloon valvuloplasty was a treatment option. Monthly cardiac evaluation was advised. It should be emphasized here that in patients with asymptomatic aortic stenosis classified as mWHO III, pregnancy is not contradicted until the symptoms occur.When the patient became pregnant, she was once monthly hospitalized in the gynecology department with cardiology consultations. During pregnancy, we did not observe progression of aortic stenosis. In the third trimester, the peak aortic gradient was even lower than in second trimester (PG max/mean 95/51 mmHg vs. 111/70 mmHg). In case of the patient, there were no reported instances of heart failure, arrythmias, or hypertension during pregnancy.

Pregnancy was terminated without cardiological complications with caesarean section in 38th week, which should be considered in all patients with aggressive aortic pathology. Due to a heart defect and full‐term pregnancy, the patient was qualified by gynecologists for caesarean section. One year after delivery, the patient was implanted with mechanical aortic valve St. Jude. Medical 21 mm with autologous pericardial patch on aortic valve sinuses with good post‐operative effects with peak aortic velocity 2.9 m/s, mean aortic gradient 19 mmHg, and peak gradient 35 mmHg. The infant did not present any symptoms of congenital heart disease, further studies confirmed this.

## 3 DISCUSSION AND CONCLUSIONS

We presented complex clinical case regarding the management of degenerative, asymptomatic severe aortic stenosis due to a congenitally bicuspid aortic valve in the setting of pregnancy. This is an area where continued addition of published data will no doubt add value. Moreover, there are no strict recommendations about the proper treatment because of the lack of clinical trials. Therefore, every single voice in discussion on the topic of cardiovascular diseases during pregnancy is of real importance.

It is well known that the natural consequence of pregnancy is an increase of the plasma volume and elevation of heart rate with concomitant decrease of the systemic vascular resistance.^3^ Even in the previously stable patient with aortic stenosis, the increased cardiac output as well as stroke volume may lead to decompensation, leading to atrial arrhythmias and heart failure development.^4^ Moreover, the sudden, massive fluid shifts that occur during delivery can be challenging for the heart.

Therefore, the decision about whether the aortic stenosis should be operated on or not should be taken with caution. Moreover, both the mechanical valve, and asymptomatic aortic stenosis qualify the patient for WHO Class III. Another difficulty is the proper timing for interventional treatment.

Current European Society of Cardiology (ESC) and the European Association for Cardio‐Thoracic Surgery (EACTS) guidelines regarding valvular heart diseases recommend intervention in asymptomatic patients who have severe aortic stenosis with concomitant left ventricle impairment (LVEF < 50%) (Class I, level B) or who develop symptoms during exercise stress test (Class I, level C).^5^ Lower level of evidence is reserved for patients with LVEF < 55% (IIa, level B) and for those with sustained decrease of blood pressure (>20 mmHg) during exercise stress test (IIa, level C). For patients with preserved LV function with LVEF > 55%, interventional treatment should be considered when the patient fulfills one of the following criteria: very severe stenosis (with peak velocity >5 m/s and mean gradient >60 mmHg), severe calcification with V max progression higher than 0.3 m/s per year, or high BNP levels in repeated measurements (>3 × corrected for age and sex normal range) (Class IIa, level B).^5^ According to these ESC/EACTS guidelines, our patient had been recommended for interventional treatment because of high mean gradient >60 mmHg with high V max. However, because of planned pregnancy, the decision about potential pros and cons of each therapeutic strategy in our patient was more complex. The recommendations presented in guidelines about pregnancy do not recommend interventional treatment for our patient. However, should the patient become symptomatic (even during an exercise stress test), intervention should be considered prior to operation.

To summarize, despite the fact that the intervention must be considered in critical stenosis with peak velocity >5 m/s and mean gradient >60 mmHg both of which this patient appears to have met, pregnancy with a mechanical valve in situ could be fraught with risk compared to pregnancy with untreated asymptomatic severe AS. That is why the intervention prior to pregnancy planning was not considered. Taking into consideration the current ESC/EACTS guidelines, the choice of a biological valve should be considered in women planning a pregnancy (class of recommendation: IIa).^5^, ^6^ Alternative approaches that may be considered include transcatheter valve implantation (especially of the pulmonary valve) and Ross surgery to treat the aortic valve.

In case of patients with a mechanical prosthesis, pregnancy is associated with a very high risk of complications.^5^, ^6^ On the other hand, both biological and mainly mechanical aortic valve replacement therapy (AVR) in pregnant patients are associated with a higher risk of cardiac and obstetric adverse events. Therefore, in each individual case, the risks should be weighed against benefits.

According to the ROPAC registry, the chance of a live birth without adverse incidents in women with a mechanical prosthesis was 58% versus 79% (*p* < 0.001) in women with a biological prosthesis and 78% (*p* < 0.001) in women with heart disease without an artificial prosthesis.^7^ Moreover, bicuspid aortic valve is associated with aortopathy, a disorder of the aorta characterized by dilataion and aneurysms, and pregnancy on its own may be combined with the increase of aortic diameter.^8^

The decision to postpone invasive treatment turned out to be right. However, we still had an alternative in the form of valvulotomy, which is a cardiac intervention performed with the balloon to open up a stiffened or stenotic valve. In patients with congenital aortic stenosis, it remains a first‐line therapeutic option for young adults and children who have stenotic valves without severe calcification.^9^ In adults, it may be considered as a bridge to decision, diagnosis, palliation, and therapy (surgical aortic valve replacement, transcatheter aortic valve replacement).^10^

The timing of potential intervention should be based on high‐risk features which we looked for. There is no exact definition of such features. However, based on available ESC guidelines, we believe that they should include: symptoms, aortic root enlargement, arrhythmias, blood pressure elevation, and instances of heart failure.^5^, ^6^

When the pregnancy was terminated with a successful delivery, the question about proper treatment came back. After confinement, patient was qualified to surgical consultation. It should be emphasized that in our patient, the treatment of choice according to cardiosurgeon team was the implantation of autologous pericardial patch on aortic valve sinuses and mechanical aortic valve due to relatively small diameter of aortic valve sinuses. Mechanical valve was chosen because of the young age of the patient and lack of further procreative plans.

Moreover, the available guidelines suggest that in some selectively chosen patients, the postponement of AVR with the possibility of close monitoring of cardiological, gynecological, and obstetric control as well as the possibility of aortic balloon angioplasty in case of emergency, seems the possible option of treatment in women with aortic stenosis planning pregnancy.^5^, ^6^, ^11^

## AUTHOR CONTRIBUTIONS

**Joanna Lewek:** Conceptualization; data curation; formal analysis; writing – original draft. **Marek Maciejewski:** Supervision; writing – review and editing. **Maciej Banach:** Supervision; writing – review and editing. **Agata Magdalena Bielecka‐Dabrowa:** Conceptualization; supervision; writing – review and editing.

## FUNDING INFORMATION

Polish Mother's Memorial Hospital Research Institute.

## CONFLICT OF INTEREST STATEMENT

None.

## CONSENT

Written informed consent was obtained from the patient to publish this report in accordance with the journal's patient consent policy.

## Data Availability

Data of the patients reported in this case are available after motivated request by email to the corresponding author.
